# *MALAT1* rs619586 as a potential genetic marker of pituitary adenoma susceptibility and aggressiveness

**DOI:** 10.3389/fendo.2026.1748441

**Published:** 2026-02-12

**Authors:** Martyna Juskiene, Monika Duseikaite-Vidike, Alvita Vilkeviciute, Ieva Baikstiene, Jurgita Makstiene, Lina Poskiene, Arimantas Tamasauskas, Rasa Verkauskiene, Rasa Liutkeviciene, Birute Zilaitiene

**Affiliations:** 1 Institute of Endocrinology, Department of Endocrinology, Lithuanian University of Health Sciences, Kaunas, Lithuania; 2 Laboratory of Ophthalmology, Institute of Neuroscience, Lithuanian University of Health Sciences, Kaunas, Lithuania; 3 Department of Pathology, Lithuanian University of Health Sciences, Kaunas, Lithuania

**Keywords:** gene variants, lncRNA, MALAT1, rs619586, rs3200401, rs1194338, pituitary adenoma

## Abstract

**Background:**

Pituitary adenomas are slow-growing tumors that originate from the anterior part of the pituitary gland. These tumors are associated with dysregulation of a number of long non-coding RNAs (lncRNAs). Metastasis-associated lung adenocarcinoma transcript-1 (*MALAT-1*) is a long non-coding RNA (lncRNA) that has been implicated in the regulation of cell proliferation, gene expression, apoptosis, differentiation, and cell cycle transition in various tumors, including pituitary adenomas (PA).

**Objective:**

To evaluate the impact of *MALAT1* gene variants (rs3200401, rs619586, and rs1194338) and immunohistochemical markers (Ki-67 and p53) on the susceptibility and clinical characteristics of PA.

**Methods:**

a case-control study included patients with PA and age- and gender-matched controls. PA diagnosis was confirmed through MRI/CT imaging and/or histopathological examination. DNA was extracted from peripheral blood samples, and three *MALAT1* variants (rs3200401, rs619586, and rs1194338) were genotyped using TaqMan^®^ real-time PCR. The expression of Ki-67 and p53 were evaluated immunohistochemically using digital image analysis. Statistical analyses included chi-square tests to compare genotype and allele distributions, logistic regression to estimate PA risk (odds ratios, 95% confidence intervals), and nonparametric tests for biomarker evaluation.

**Results:**

Among 390 participants (145 PA and 245 controls), only the *MALAT1* rs619586 variant showed statistically significant associations after Bonferroni correction (p < 0.016). The rs619586 G allele was more frequent in PA patients than in controls (4.1% vs. 0.8%, p = 0.001) and increased the odds of developing PA by 4.1-fold under the additive model (OR = 4.139, 95% CI: 1.365- 12.551, p = 0.012). The G allele remained significantly associated across several clinical subgroups, including microadenomas, macroadenomas, invasive PAs, and PAs with recurrence (p ≤ 0.015). In PA tissues, p53 H-scores were higher in macroadenomas compared with microadenomas (p = 0.047), and patients with the rs619586 AA genotype showed significantly higher p53 expression than those with the AG genotype (p = 0.008). A moderate positive correlation was observed between Ki-67 LI and p53 expression (ρ = 0.268, p = 0.035).

**Conclusions:**

*MALAT1* rs619586 G allele is significantly associated with an increased risk of PA and its more aggressive clinical features, including invasiveness and recurrence. These findings suggest that rs619586 may serve as a potential genetic marker linked to PA susceptibility. Additionally, the observed relationship between p53 expression and tumor proliferation highlights its potential role in PA tumorigenesis. Further studies are needed to confirm these associations and clarify the underlying molecular mechanisms.

## 1 Introduction

Pituitary adenomas (PAs) are among the most prevalent benign intracranial tumors, originating from the adenohypophyseal cells of the anterior pituitary gland. They constitute approximately 10- 15% of all surgically resected intracranial tumors, making them a significant clinical entity within neuro-oncology and endocrinology (1, 2). Epidemiological studies indicate that PAs are common in the general population, with prevalence rates reaching up to 20% based on autopsy and radiological findings (3). The tumors present a broad spectrum of clinical manifestations, primarily determined by their size and hormonal activity. Microadenomas are defined as tumors smaller than 1cm, often identified incidentally or during evaluation for endocrine dysfunction, while macroadenomas exceed 1 cm and are more likely to cause compressive symptoms such as headaches, visual disturbances, and hypopituitarism. Moreover, PAs are classified based on their hormonal secretion into functioning and non-functioning adenomas. Functioning adenomas secrete excess hormones like prolactin, growth hormone (GH), adrenocorticotropic hormone (ACTH), and others, leading to clinical syndromes such as hyperprolactinemia, acromegaly, and Cushing’s disease. Conversely, non-functioning adenomas may remain asymptomatic until they grow sufficiently large to induce compressive effects (3–5). Despite their benign histology, PAs display a capacity for invasive growth, with approximately 45-55% invading local structures such as the cavernous sinus, sphenoid sinus, or dura mater. This local invasion complicates surgical resection and increases the likelihood of residual disease and recurrence. Residual disease and recurrence are clinically significant, as they often necessitate repeated surgical interventions, long-term medical management, and radiotherapy, thereby increasing patient morbidity, diminishing quality of life, and raising healthcare costs. Accurate assessment of invasiveness and tumor behavior remains challenging, as traditional histopathological markers, like Ki-67 proliferation index and p53 expression, lack sufficient predictive power. Hence, there is a pressing need to identify reliable biomarkers that can facilitate early diagnosis, predict tumor aggressiveness, and guide personalized treatment strategies (6).

Recent advances in molecular biology have revolutionized our understanding of tumorigenesis, emphasizing the pivotal roles of genetic and epigenetic factors beyond classical oncogenes and tumor suppressor genes. Notably, non-coding RNAs (ncRNAs), including microRNAs (miRNAs) and long non-coding RNAs (lncRNAs), have emerged as critical regulators of gene expression. These molecules, although not translating into proteins, influence cellular processes such as proliferation, apoptosis, differentiation, and metastasis. In particular, lncRNAs, which are transcripts longer than 200 nucleotides, have been implicated in tumor initiation and progression across various cancer types, including neuroendocrine tumors such as pituitary adenoma, papillary thyroid carcinoma and gastrointestinal neuroendocrine tumors (7–9).

Metastasis-associated lung adenocarcinoma transcript 1 (*MALAT1*) lncRNA, which was initially identified as an oncogene in a study of non-small cell lung cancer (NSCLC), is situated at 11q13 (9). Since its discovery, *MALAT1* has contributed significantly to the progression, metastasis, drug resistance, and treatment of cancer, as well as its clinical importance in predicting the tumor metastasis of early-stage cancer, particularly lung cancer (9, 10). Subsequently, overexpression of *MALAT1* was also found to be involved in tumor cell proliferation, migration, invasion, and apoptosis in various cancers. In addition, increased expression level of *MALAT1* can also be used as a potential biomarker for tumor diagnosis and prognosis, including liver, colorectal, pancreatic, papillary thyroid, renal cancers, and gastrointestinal diffuse large B-cell lymphoma (11–16).

In addition to *MALAT1*, other molecular markers such as Ki-67, p53 are being explored to improve diagnostic accuracy and prognostic predictions. Ki-67, a nuclear protein associated with cellular proliferation, has been correlated with tumor aggressiveness, although its standalone utility remains limited. Mutations and expression patterns of p53, a tumor suppressor, are also under investigation, yet their roles in PA are not fully clarified. Given the complex molecular landscape of PAs, integrating genetic, epigenetic, and molecular profiles could enhance our ability to stratify tumors based on their invasive potential and risk of recurrence. Specifically, the study of lncRNA gene variants, such as those in *MALAT1*, may unearth novel insights into tumor biology and facilitate the development of targeted therapies in patients with PA.

## 2 Materials and methods

### 2.1 Study design

The research was carried out at the Institute and Department of Endocrinology, the Institute of Neuroscience, the Laboratory of Ophthalmology, and the Department of Pathology of the Lithuanian University of Health Sciences (LUHS) Hospital Kauno Klinikos. Ethical approval was granted by the Kaunas Regional Biomedical Research Ethics Committee (No. BE-2-47, issued on December 25, 2016). All participants were fully informed about the study’s purpose and procedures, and each provided written consent in compliance with ethical research standards.

### 2.2 Study population

The study group consisted of 145 patients diagnosed with pituitary adenoma (PA), while the control group included 245 healthy individuals. Patient selection was conducted according to predefined inclusion and exclusion criteria. The median age of PA patients was 53 years (IQR = 20), whereas the median age of the control group was 55 years (IQR = 22). Age and gender distributions did not differ significantly between the groups (p > 0.05), and the control group was therefore considered adequately matched to the PA group. The detailed inclusion and exclusion criteria have been described in our previous work (17). Although control subjects were not systematically screened with pituitary imaging, incidental microadenomas are common and usually clinically silent, while clinically significant PAs are rare. Any resulting non-differential misclassification would therefore be expected to attenuate, rather than inflate, the observed genetic associations.

At the available sample size (145 cases and 245 controls) and α=0.016, *post-hoc* power indicates adequate power (≥80%) only for detecting comparatively large effects (approximately OR ≥ 2 for rs1194338/rs3200401, and OR ≥ 9 for rs619586). Therefore, analyses involving rs619586, particularly subgroup stratifications, should be interpreted as exploratory and require replication in larger cohorts.

All participants were recruited from the same geographic region (Lithuania) and represent a relatively homogeneous Northern European population. Cases and controls were matched by age and sex and were enrolled at the same tertiary referral center. Although ancestry informative markers were not analyzed, all genotype distributions in the control group conformed to Hardy–Weinberg equilibrium, reducing the likelihood of major population stratification effects.

### 2.3 SNV selection criteria

The *MALAT1* SNVs (rs1194338, rs619586, and rs3200401) were chosen because they are the most widely studied *MALAT1* variants. Many studies across different cancer types have explored their effects on *MALAT1* expression and tumor behavior.

In most malignancies, the rs619586 G allele confers protection: in papillary thyroid cancer it decreases susceptibility and down-regulates MALAT1, reducing proliferation and promoting apoptosis (18); in meningioma, the G allele reduces invasiveness by down-regulating COL5A1 (19); and in neuroblastoma, it is linked to reduced risk and increased NEAT1 expression (20). By contrast, in oral squamous cell carcinoma *MALAT1* gene single nucleotide variant rs619586 (AG/GG genotype) is associated with a more advanced stage and larger tumour size (21).

Evidence for rs3200401 is inconsistent: meta-analyses report no overall effect on cancer risk, although a modest increase in colorectal cancer risk has been noted (22), and a Korean study found that CT, CT+TT genotypes increase gastric cancer risk in certain subgroups (23).

Regarding the *MALAT1* single nucleotide variant rs1194338, the majority of published studies suggest that this variant confers a protective effect against cancer development and tumor aggressiveness, but not universally across all tumor types. In colorectal cancer, the A allele correlates with lower MALAT1 expression and less advanced disease (24). In hepatocellular carcinoma, carriers of the CA or AA genotype correlate with a lower risk of vascular invasion and severe disease (25).

To our knowledge, however, no previous work has examined these variants in PA. Our study fills this gap by demonstrating that, unlike in several other tumour types where rs619586 is protective, the rs619586 G allele is associated with increased risk and aggressiveness in PA.

### 2.4 Activeness, recurrence, and invasiveness evaluation

The analysis of all PAs was based on histopathological findings of the tumor and preoperative hormone levels in the blood serum. All 145 subjects were categorized into two groups: hormonally active and inactive PA. The hormonally active PA group was not further subdivided according to specific hormone secretion because the majority of tumors were prolactinomas (i.e., prolactin-secreting pituitary adenomas), and the remaining functioning subtypes were insufficient in number for meaningful subgroup analysis. Since some of the 145 subjects had previously undergone surgery, patients were additionally categorized by recurrence of PA into two groups: PA with and without recurrence. Recurrence was defined as enlargement of a residual tumor or the appearance of a new growth on follow-up magnetic resonance imaging (MRI) after surgical resection. The residual tumor was considered stable if there were no signs of tumor progression on follow-up MRI. Most prolactinomas were surgically treated because of the remaining pressure effects of surrounding structures or because of ineffective medical treatment.

Recurrence was defined radiologically as enlargement of residual tumor tissue or the appearance of new tumor growth on follow-up magnetic resonance imaging after surgical treatment. Due to the retrospective design and limited availability of detailed surgical extent-of-resection data, distinction between true biological recurrence and regrowth related to incomplete resection was not feasible.

Tumor invasiveness was assessed preoperatively using magnetic resonance imaging (MRI) and classified according to the Knosp grading system by an experienced neuroradiologist. Tumors graded as Knosp 3B–4 were considered invasive, whereas grades 0–3A were classified as non-invasive. Although Knosp grading is the standard radiological method for evaluating cavernous sinus invasion and guiding surgical planning, it does not fully capture microscopic or histologically confirmed invasion.

### 2.5 DNA extraction, Genotyping, and Immunohistochemistry

The genotyping of *MALAT1* lncRNA (rs3200401, rs619586, and rs1194338) was performed at the Laboratory of Ophthalmology, Neuroscience Institute, Lithuanian University of Health Sciences (LUHS).

DNA extraction, single-nucleotide variant genotyping, and immunohistochemistry analysis were performed as previously described in our earlier publication (17). In accordance with World Health Organization and European Society of Endocrinology guidelines, immunohistochemical evaluation in our centre routinely includes Ki-67 and p53 as established prognostic markers. Other markers, such as PD-L1 and cyclin D1, are currently under investigation but are not part of our routine diagnostic panel and were therefore not included in the present study.

#### 2.5.1 Genotyping quality control

Genotyping was performed using TaqMan^®^ allelic discrimination assays according to the manufacturer’s instructions. Samples with failed or ambiguous genotype calls were excluded prior to analysis, resulting in a complete call rate for all analyzed variants. Genotype clusters were visually inspected to confirm clear allele separation. Blind duplicate samples were included as an internal quality control measure, with 100% concordance observed between duplicates. Genotype distributions for all variants were consistent with Hardy–Weinberg equilibrium in the control group, supporting genotyping accuracy.

### 2.6 Statistical analysis

Statistical analysis was performed using the SPSS/W 31 software (Statistical Package for the Social Sciences for Windows, Inc., Chicago, IL, USA). Descriptive variables are presented as absolute numbers and percentages, while continuous data are expressed as medians with interquartile ranges (IQRs). Genotypic and allelic distributions between patients with PA and control subjects were compared using the chi-square test. The relationship between *MALAT1* genotypes and the risk of PA development was evaluated through binary logistic regression, with results expressed as odds ratios (ORs) and 95% confidence intervals (CIs). The most suitable genetic model was determined according to the Akaike Information Criterion (AIC), with lower AIC values. For immunohistochemical markers, nonparametric statistical methods were applied. The Mann-Whitney U test was used to compare p53 H-scores across PA subgroups, and the correlation between the Ki-67 LI and p53 H-score was analyzed using Spearman’s rank-order correlation coefficient (ρ). Although analyses were restricted to predefined SNVs and markers based on specific hypotheses, Bonferroni correction was applied, and statistical significance was set at *p* < 0.016.

## 3 Results

A case-control study was conducted involving 390 subjects divided into two groups: the control group (n = 245) and a group of PA (n = 145). After forming the groups of subjects, *MALAT1* rs1194338, rs619586, and rs3200401 variants were analyzed. The median age of PA patients was 53 years. The patients’ group was later divided into subgroups by the PA’s tumor size, hormonal activity, invasiveness, and recurrence. The median age of the control group was 55 years. The age and gender did not differ between study groups (p > 0.05). The demographic data of the subjects are presented in **Table 1**.

**Table 1:** Demographic characteristics of study subjects.

Characteristics	Group		*P*-value
	PA, n (%) (n = 145)	Control, n (%) (n = 245)	
Age median (IQR)	53 (20)	55 (22)	0.600*
Gender, n % Females Males	80 (55.2) 65 (44.8)	135 (55.1) 110 (44.9)	0.989**
Tumor size, n (%) Micro PA Macro PA	45 (31) 100 (69)	–	–
Hormonal activity, n (%) Active Non-active	79 (54.5) 66 (45.5)	–	–
Invasiveness, n (%) Invasive Non-invasive	75 (51.7) 70 (48.3)	–	–
Recurrence, n (%) PA without recurrence PA with recurrence	116 (80) 29 (20)	–	–

*Mann-Whitney U test; **Pearson Chi-Square test

We examined the genotype and allele frequency distributions of *MALAT1* rs1194338, rs619586, and rs3200401 in the PA and the control groups. The analysis showed that the *MALAT1* rs619586 G allele is statistically significantly more frequent in PA patients than in the control group subjects (4.1% vs. 0.8%, p = 0.001) (**Table 2**).

**Table 2:** Distributions of *MALAT1* (rs1194338, rs619586, and rs3200401) genotypes and alleles in patients with PA and control groups.

Gene	Genotype/Allele	PA group n (%) (n=145)	Control group n (%) (n=245)	*P*-value	HWE *P-*value
*MALAT1* (rs1194338)	CC	91 (62.8)	145 (59.2)	0.629	0.592
	CA	48 (33.1)	85 (34.7)		
	AA	6 (4.1)	15 (6.1)		
	In total:	145 (100)	245 (100)		
	Allele: C A	230 (79.3) 60 (20.7)	375 (76.5) 115 (23.5)	0.368	
*MALAT1* (rs619586)	AA	135 (93.1)	241 (98.4)	0.017	0.897
	AG	8 (5.5)	4 (1.6)		
	GG	2 (1.4)	0 (0)		
	In total:	145 (100)	245 (100)		
	Allele: A G	278 (95.9) 12 (4.1)	486 (99.2) 4 (0.8)	**0.001**	
*MALAT1* (rs3200401)	CC	101 (69.7)	175 (71.4)	0.770	0.213
	CT	40 (27.6)	61 (24.9)		
	TT	4 (2.8)	9 (3.7)		
	In total:	145 (100)	245 (100)		
	Allele: C T	242 (83.4) 48 (16.6)	411 (83.9) 79 (16.1)	0.875	

Bold p-values indicate statistical significance after Bonferroni correction (p < 0.016).

To assess the impact of *MALAT1* gene variants on the onset of PA, we conducted a binary logistic regression analysis (**Table 3**). The statistically significant findings revealed that the G allele of *MALAT1* rs619586 increases the odds of developing PA by 4.1-fold under the additive model (OR= 4.139, 95% CI: 1.365-12.551, p = 0.012).

**Table 3:** Binary logistic regression analysis of *MALAT1* (rs1194338, rs619586, and rs3200401) in patients with PA and control groups.

*MALAT1* (rs1194338)				
Model	Genotype/Allele	OR (95% CI)	*P*-value	AIC
Codominant	CA vs. CC AA vs. CC	0.900 (0.579-1.398) 0.637 (0.239-1.702)	0.638 0.369	517.774
Dominant	CA+AA vs. CC	0.860 (0.564-1.312)	0.485	516.236
Recessive	AA vs. CC+CA	0.662 (0.251-1.746)	0.404	515.995
Overdominant	CA vs. CC+AA	0.931 (0.603-1.438)	0.749	516.622
Additive	A	0.853 (0.601-1.210)	0.374	515.925
*MALAT1* (rs619586)				
Model	Genotype/Allele	OR (95% CI)	*P*-value	AIC
Codominant	AG vs. AA GG vs. AA	3.570 (1.056-12.076) -	0.041 -	510.231
Dominant	AG+GG vs. AA	4.463 (1.373-14.503)	0.013	509.706
Recessive	GG vs. AA+AG	–	–	512.750
Overdominant	AG vs. AA+GG	3.518 (1.040-11.898)	0.043	512.309
Additive	G	4.139 (1.365-12.551)	**0.012**	**508.684**
*MALAT1* (rs3200401)				
Model	Genotype/Allele	OR (95% CI)	*P*-value	AIC
Condominant	CT vs. CC TT vs. CC	1.136 (0.712-1.814) 0.770 (0.231-2.564)	0.593 0.670	518.198
Dominant	CT+TT vs. CC	1.089 (0.695-1.707)	0.710	516.587
Recessive	TT vs. CC+CT	0.744 (0.225-2.460)	0.628	516.482
Overdominant	CT vs. CC+TT	1.149 (0.722-1.830)	0.558	516.348
Additive	T	1.030 (0.703-1.511)	0.878	516.702

OR, odds ratio; CI, confidence interval; AIC, Akaike information criteria; p-value, significance level (statistically significant when p < 0.016).

Bold p-values indicate statistical significance after Bonferroni correction (p < 0.016). Bold AIC values indicate the best-fitting model (AIC).

### 3.1 Gender-based analysis

When stratified by gender, no statistically significant differences in the distribution of *MALAT1* genotypes or alleles were observed among female participants (**Supplementary Table S1** and **Supplementary Table S2**). However, in the male subgroup, the analysis showed that the *MALAT1* rs619586 G allele was found to be statistically significantly more frequent in patients with PA compared to male controls (5.4% vs. 0.9%, p = 0.010) (**Table 4**).

**Table 4:** Distributions of *MALAT1* (rs1194338, rs619586, and rs3200401) genotypes and alleles in male patients with PA and the control group.

Gene	Genotype/Allele	PA group males (n=65) n (%)	Control group males (n=110) n (%)	*P*-value
*MALAT1* (rs1194338)	CC	45 (69.2)	66 (60)	0.467
	CA	18 (27.7)	39 (35.5)	
	AA	2 (3.1)	5 (4.5)	
	In total:	65 (100)	110 (100)	
	Allele: C A	108 (83.1) 22 (16.9)	171 (77.7) 49 (22.3)	0.229
*MALAT1* (rs619586)	AA	60 (92.3)	108 (98.2)	0.098
	AG	3 (4.6)	2 (1.8)	
	GG	2 (3.1)	0 (0)	
	In total:	65 (100)	110 (100)	
	Allele: A G	123 (94.6) 7 (5.4)	218 (99.1) 2 (0.9)	**0.010**
*MALAT1* (rs3200401)	CC	49 (75.4)	82 (74.5)	0.399
	CT	16 (24.6)	25 (22.7)	
	TT	0 (0)	3 (2.7)	
	In total:	65 (100)	110 (100)	
	Allele: C T	114 (87.7) 16 (12.3)	189 (85.9) 31 (14.1)	0.636

Bold p-values indicate statistical significance after Bonferroni correction (p < 0.016).

Binary logistic regression analysis revealed no statistically significant differences between males with PA and the control group males (all p > 0.016) (**Supplementary Table S3**).

The overall sex distribution did not differ significantly between PA patients and controls, indicating no clear gender predominance in pituitary adenoma occurrence in this cohort. Although a higher frequency of the rs619586 G allele was observed among male PA patients at the allele level, this finding did not translate into a statistically significant association in corrected regression analyses and should therefore be interpreted with caution.

### 3.2 Associations of *MALAT1* (rs1194338, rs619586 and rs3200401) with pituitary adenoma’s tumor size

PA was divided into Micro PA and Macro PA groups. After evaluating the distribution of genotypes and alleles of *MALAT1* rs1194338, rs619586, and rs3200401 variants in Micro/Macro PA and the control groups, statistically significant differences in the distribution of *MALAT1* rs619586 genotypes (AA, AG, and GG) were found between Micro PA and controls: 91.1%, 8.9% and 0% vs. 98.4%, 1.6% and 0%, respectively (p=0.006). The same variant G allele was also more frequent in the Micro PA group than in the control group (4.4% vs. 0.8%, p = 0.006). Also, in the Macro PA group, it was found that the *MALAT1* rs619586 G allele was more frequent in the Macro PA group than in the control group (4% vs. 0.8%, p = 0.003) (**Table 5**).

**Table 5:** Distributions of *MALAT1* (rs1194338, rs619586, and rs3200401) genotypes and alleles in PA and control groups by PA tumor size.

Gene	Genotype/Allele	Control group (n=245) n (%)	Micro PA (n=45) n (%)	*P*-value	Macro PA (n=100) n (%)	*P*-value
*MALAT1* (rs1194338)	CC	145 (59.2)	33 (73.3)	0.174	58 (58)	0.870
	CA	85 (34.7)	11 (24.4)		37 (37)	
	AA	15 (6.1)	1 (2.2)		5 (5)	
	In total:	245 (100)	45 (100)		100 (100)	
	Allele: C A	375 (76.5) 115 (23.5)	77 (85.6) 13 (14.4)	0.057	153 (76.5) 47 (23.5)	0.993
*MALAT1* (rs619586)	AA	241 (98.4)	41 (91.1)	**0.006**	94 (94)	0.034
	AG	4 (1.6)	4 (8.9)		4 (4)	
	GG	0 (0)	0 (0)		2 (2)	
	In total:	245 (100)	45 (100)		100 (100)	
	Allele: A G	486 (99.2) 4 (0.8)	86 (95.6) 4 (4.4)	**0.006**	192 (96) 8 (4)	**0.003**
*MALAT1* (rs320040)1)	CC	175 (71.4)	33 (73.3)	0.909	68 (68)	0.486
	CT	61 (24.9)	10 (22.2)		30 (30)	
	TT	9 (3.7)	2 (4.4)		2 (2)	
	In total:	245 (100)	45 (100)		100 (100)	
	Allele: C T	411 (83.9) 79 (16.1)	76 (84.4) 14 (15.6)	0.892	166 (83) 34 (17)	0.777

Bold p-values indicate statistical significance after Bonferroni correction (p < 0.016).

Binary logistic regression analysis revealed that the G allele of *MALAT1* rs619586 increases the odds of developing Micro PA by 5.8-fold under the additive model (OR = 5.878, 95% CI: 1.414- 24.438, p = 0.015) (**Table 6**).

**Table 6:** Binary logistic regression analysis of *MALAT1* (rs1194338, rs619586, and rs3200401) in the PA and control groups by PA tumor size.

*MALAT1* (1194338)				
Model	Genotype/Allele	OR (95% CI)	*P*-value	AIC
Micro PA				
Codominant	CA vs. CC AA vs. CC	0.569 (0.273-1.184) 0.293 (0.037-2.297)	0.131 0.243	250.524
Dominant	CA+AA vs. CC	0.527 (0.260-1.070)	0.076	248.965
Recessive	AA vs. CC+CA	0.348 (0.045-2.706)	0.313	250.950
Overdominant	CA vs. CC+AA	0.609 (0.294-1.262)	0.182	250.431
Additive	A	0.559 (0.301-1.039)	0.066	248.531
Macro PA				
Codominant	CA vs. CC AA vs. CC	1. 088 (0.666-1.779) 0.833 (0.290-2.398)	0.736 0.735	419.113
Dominant	CA+AA vs. CC	1.050 (0.655-1.683)	0.839	417.354
Recessive	AA vs. CC+CA	0.807 (0.285-2.283)	0.686	417.227
Overdominant	CA vs. CC+AA	1.106 (0.682-1.793)	0.684	417.231
Additive	A	1.002 (0.682-1.472)	0.993	417.395
*MALAT1* (rs619586)				
Model	Genotype/Allele	OR (95% CI)	*P*-value	AIC
Micro PA				
Codominant	AG vs. AA GG vs. AA	5.878 (1.414-24.438) -	0.015 -	248.941
Dominant	AG+GG vs. AA	5.878 (1.414-24.438)	0.015	246.941
Recessive	GG vs. AA+AG	–	–	252.315
Overdominant	AG vs. AA+GG	5.878 (1.414-24.438)	0.015	246.941
Additive	G	5.878 (1.414-24.438)	**0.015**	**246.941**
Macro PA				
Codominant	AG vs. AA GG vs. AA	2.564 (0.628-10.462) -	0.189 -	412.746
Dominant	AG+GG vs. AA	3.846 (1.061-13.935)	0.040	413.116
Recessive	GG vs. AA+AG	–	–	412.413
Overdominant	AG vs. AA+GG	2.510 (0.615-10.241)	0.199	415.799
Additive	G	3.601 (1.153-11.245)	0.027	411.696
*MALAT1* (rs3200401)				
Micro PA				
Model	Genotype/Allele	OR (95% CI)	*P*-value	AIC
Codominant	CT vs. CC TT vs. CC	0.869 (0.404-1.869) 1.178 (0.244-5.702)	0.720 0.838	254.125
Dominant	CT+TT vs. CC	0.909 (0.444-1.861)	0.794	252.246
Recessive	TT vs. CC+CT	1.220 (0.255-5.840)	0.804	252.255
Overdominant	CT vs. CC+TT	0.862 (0.403-1.843)	0.701	252.165
Additive	T	0.962 (0.532-1.740)	0.897	252.298
Macro PA				
Codominant	CT vs. CC TT vs. CC	1.266 (0.753-2.127) 0.572 (0.120-2.715)	0.374 0.482	417.909
Dominant	CT+TT vs. CC	1.176 (0.711-1.946)	0.527	416.998
Recessive	TT vs. CC+CT	0.535 (0.114-2.522)	0.429	416.691
Overdominant	CT vs. CC+TT	1.293 (0.771-2.167)	0.330	416.458
Additive	T	1.063 (0.690-1.639)	0.781	417.319

OR, odds ratio; CI, confidence interval; AIC, Akaike information criteria; p-value: significance level (statistically significant when p < 0.016).

Bold p-values indicate statistical significance after Bonferroni correction (p < 0.016). Bold AIC values indicate the best-fitting model (AIC).

### 3.2 Associations of *MALAT1* (rs1194338, rs619586 and rs3200401) with pituitary adenoma’s invasiveness

Distribution of the genotypes and alleles’ analysis was performed between the non-invasive and invasive PA groups and the control group. After evaluating the distribution of genotypes and alleles of *MALAT1* rs1194338, rs619586, and rs3200401 variants in non-invasive/invasive PA and the control groups, the analysis revealed that the *MALAT1* rs619586 G allele was statistically significantly more frequent in the non-invasive PA group than in the control group subjects (3.6% vs. 0.8%, p = 0.015). In the invasive PA group, our statistical analysis revealed that the same variant genotype distributions (AA, AG, and GG) differ between the invasive PA and control groups (92%, 6.7%, and 1.3% vs. 98.4%, 1.6%, and 0%, p = 0.013). Also, the analysis revealed that the *MALAT1* rs619586 G allele was statistically significantly more frequent in the invasive PA group than in the control group subjects (4.7% vs. 0.8%, p = 0.001) (**Table 7**).

**Table 7:** Distributions of *MALAT1* (rs1194338, rs619586, and rs3200401) genotypes and alleles in PA and control groups by PA invasiveness.

Gene	Genotype/Allele	Control group (n=245) n (%)	Non- invasive PA (n=70) n (%)	*P*-value	Invasive PA (n=75) n (%)	*P*-value
*MALAT1* (rs1194338)	CC	145 (59.2)	46 (65.7)	0.443	45 (60)	0.967
	CA	85 (34.7)	22 (31.4)		26 (34.7)	
	AA	15 (6.1)	2 (2.9)		4 (5.3)	
	In total:	245 (100)	70 (100)		75 (100)	
	Allele: C A	375 (76.5) 115 (23.5)	114 (81.4) 26 (18.6)	0.220	116 (77.3) 34 (22.7)	0.838
*MALAT1* (rs619586)	AA	241 (98.4)	66 (94.3)	0.070	69 (92)	**0.013**
	AG	4 (1.6)	3 (4.3)		5 (6.7)	
	GG	0 (0)	1 (1.4)		1 (1.3)	
	In total:	245 (100)	70 (100)		75 (100)	
	Allele: A G	486 (99.2) 4 (0.8)	135 (96.4) 5 (3.6)	**0.015**	143 (95.3) 7 (4.7)	**0.001**
*MALAT1* (rs3200401)	CC	175 (71.4)	49 (70)	0.558	52 (69.3)	0.940
	CT	61 (24.9)	20 (28.6)		20 (26.7)	
	TT	9 (3.7)	1 (1.4)		3 (4)	
	In total:	245 (100)	70 (100)		75 (100)	
	Allele: C T	411 (83.9) 79 (16.1)	118 (84.3) 22 (15.7)	0.907	124 (82.7) 26 (17.3)	0.726

Bold p-values indicate statistical significance after Bonferroni correction (p < 0.016).

Binary logistic regression analysis revealed that the G allele of *MALAT1* rs619586 increases the odds of developing invasive PA by 4.9-fold under the additive model (OR = 4.910, 95% CI: 1.430- 16.851, p = 0.011) (**Table 8**).

**Table 8:** Binary logistic regression analysis of *MALAT1* (rs1194338, rs619586, and rs3200401) in the PA and control groups by PA invasiveness.

*MALAT1* (1194338)				
Non-invasive PA				
Model	Genotype/Allele	OR (95% CI)	*P*-value	AIC
Codominant	CA vs. CC AA vs. CC	0.816 (0.459-1.449) 0.420 (0.093-1.907)	0.487 0.261	335.925
Dominant	CA+AA vs. CC	0.757 (0.434-1.318)	0.325	334.730
Recessive	AA vs. CC+CA	0.451 (0.101-2.021)	0.298	334.413
Overdominant	CA vs. CC+AA	0.863 (0.488-1.524)	0.611	335.454
Additive	A	0.748 (0.467-1.198)	0.227	334.194
Invasive PA				
Codominant	CA vs. CC AA vs. CC	0.986 (0.567-1.712) 0.859 (0.271-2.721)	0.959 0.796	352.418
Dominant	CA+AA vs. CC	0.967 (0.570-1.638)	0.900	350.470
Recessive	AA vs. CC+CA	0.864 (0.278-2.686)	0.800	350.420
Overdominant	CA vs. CC+AA	0.999 (0.580-1.720)	0.997	350.486
Additive	A	0.957 (0.623-1.470)	0.841	350.445

*MALAT1* (rs619586)				
Non-invasive PA				
Model	Genotype/Allele	OR (95% CI)	*P*-value	AIC
Codominant	AG vs. AA GG vs. AA	2.739 (0.598-12.541) -	0.194 -	333.139
Dominant	AG+GG vs. AA	3.652 (0.889-14.992)	0.072	332.668
Recessive	GG vs. AA+AG	–	–	333.696
Overdominant	AG vs. AA+GG	2.698 (0.589-12.350)	0.201	334.201
Additive	G	3.561 (0.995-12.743)	0.051	331.763
Invasive PA				
Codominant	AG vs. AA GG vs. AA	4.366 (1.141-16.703) -	0.031 -	345.061
Dominant	AG+GG vs. AA	5.239 (1.438-19.093)	0.012	344.156
Recessive	GG vs. AA+AG	–	–	347.574
Overdominant	AG vs. AA+GG	4.304 (1.125-16.459)	0.033	346.055
Additive	G	4.910 (1.430-16.851)	**0.011**	**343.362**

*MALAT1*(rs3200401)				
Non-invasive PA				
Model	Genotype/Allele	OR (95% CI)	*P*-value	AIC
Codominant	CT vs. CC TT vs. CC	1.171 (0.645-2.125) 0.397 (0.049-3.209)	0.604 0.386	336.390
Dominant	CT+TT vs. CC	1.071 (0.599-1.917)	0.816	335.661
Recessive	TT vs. CC+CT	0.380 (0.047-3.052)	0.363	334.657
Overdominant	CT vs. CC+TT	1.207 (0.666-2.185)	0.535	335.336
Additive	T	0.971 (0.587-1.607)	0.910	335.702
Invasive PA				
Codominant	CT vs. CC TT vs. CC	1.103 (0.610-1.995) 1.122 (0.293-4.296)	0.745 0.867	352.364
Dominant	CT+TT vs. CC	1.106 (0.629-1.943)	0.727	350.364
Recessive	TT vs. CC+CT	1.093 (0.288-4.144)	0.896	350.469
Overdominant	CT vs. CC+TT	1.097 (0.609-1.975)	0.758	350.391
Additive	T	1.084 (0.679-1.732)	0.736	350.373

OR, odds ratio; CI, confidence interval; AIC, Akaike information criteria; p-value: significance level (statistically significant when p < 0.016).

Bold p-values indicate statistical significance after Bonferroni correction (p < 0.016). Bold AIC values indicate the best-fitting model (AIC).

### 3.3 Associations of *MALAT1* (rs1194338, rs619586 and rs320040) with Pituitary Adenomas’ activity

The frequencies of genotypes and alleles for the selected single nucleotide variants (SNVs) were analyzed within the study groups, stratified by PAs’ activeness. In both the non-active and active PA subgroups, the analysis showed that the *MALAT1* rs619586 G allele was statistically significantly more frequent in PA subgroups than in the control group (4.5% vs. 0.8%, p = 0.002; 3.8% vs. 0.8%, p = 0.008, respectively) (**Table 9**).

**Table 9:** Distributions of *MALAT1* (rs1194338, rs619586, and rs3200401) genotypes and alleles in patients with PA and control groups by PA activity.

Gene	Genotype/Allele	Control group (n=245) n (%)	Non- active PA (n=66) n (%)	*P*-value	Active PA (n=79) n (%)	*P*-value
*MALAT1* (rs1194338)	CC	145 (59.2)	44 (66.7)	0.431	47 (59.5)	0.939
	CA	85 (34.7)	20 (30.3)		28 (35.4)	
	AA	15 (6.1)	2 (3)		4 (5.1)	
	In total:	245 (100)	66 (100)		79 (100)	
	Allele: C A	375 (76.5) 115 (23.5)	108 (81.8) 24 (18.2)	0.195	122 (77.2) 36 (22.8)	0.859
*MALAT1* (rs619586)	AA	241 (98.4)	61 (92.4)	0.020	74 (93.7)	0.048
	AG	4 (1.6)	4 (6.1)		4 (5.1)	
	GG	0 (0)	1 (1.5)		1 (1.3)	
	In total:	245 (100)	66 (100)		79 (100)	
	Allele: A G	486 (99.2) 4 (0.8)	126 (95.5) 6 (4.5)	**0.002**	152 (96.2) 6 (3.8)	**0.008**
*MALAT1* (rs3200401)	CC	175 (71.4)	52 (78.8)	0.421	49 (62)	0.264
	CT	61 (24.9)	13 (19.7)		27 (34.2)	
	TT	9 (3.7)	1 (1.5)		3 (3.8)	
	In total:	245 (100)	66 (100)		79 (100)	
	Allele: C T	411 (83.9) 79 (16.1)	117 (88.6) 15 (11.4)	0.175	125 (79.1) 33 (20.9)	0.168

Bold p-values indicate statistical significance after Bonferroni correction (p < 0.016).

Binary logistic regression analysis between the non-active/active PA group and the control group of *MALAT1* rs1194338, rs619586, and rs3200401 did not show any statistically significant results (**Supplementary Table S4**).

### 3.4 Associations of *MALAT1* (rs1194338, rs619586 and rs3200401) with Pituitary Adenomas’ recurrence

All patients with PA were also divided into PA without recurrence and PA with recurrence groups. After evaluating the distribution of genotypes and alleles of *MALAT1* rs1194338, rs619586, and rs3200401 variants in PA without recurrence/PA with recurrence and the control group, the analysis revealed that the *MALAT1* rs619586 G allele was statistically significantly more frequent in the non-invasive PA group than in the control group subjects (3.9% vs. 0.8%, p = 0.003). In the PA with recurrence group, it was found that the same variant genotype distributions (AA, AG, and GG) differ between the PA with recurrence group and control group (89.7%, 10.3%, and 0% vs. 98.4%, 1.6%, and 0%, p = 0.005). Also, the analysis revealed that the *MALAT1* rs619586 G allele was statistically significantly more frequent in the PA with recurrence group than in the control group subjects (5.2% vs. 0.8%, p = 0.005) (**Table 10**).

**Table 10:** Distributions of *MALAT1* (rs1194338, rs619586, and rs3200401) genotypes and alleles in patients with PA and control groups by PA recurrence.

Gene	Genotype/Allele	Control group (n=245) n (%)	PA without recurrence (n=116) n (%)	*P*-value	PA with recurrence (n=29) n (%)	*P*-value
*MALAT1* (rs1194338)	CC	145 (59.2)	76 (65.5)	0.387	15 (51.7)	0.741
	CA	85 (34.7)	36 (31)		12 (41.4)	
	AA	15 (6.1)	4 (3.4)		2 (6.9)	
	In total:	245 (100)	116 (100)		29 (100)	
	Allele: C A	375 (76.5) 115 (23.5)	188 (81) 44 (19)	0.172	42 (72.4) 16 (27.6)	0.486
*MALAT1* (rs619586)	AA	241 (98.4)	109 (94)	0.036	26 (89.7)	**0.005**
	AG	4 (1.6)	5 (4.3)		3 (10.3)	
	GG	0 (0)	2 (1.7)		0 (0)	
	In total:	245 (100)	116 (100)		29 (100)	
	Allele: A G	486 (99.2) 4 (0.8)	223 (96.1) 9 (3.9)	**0.003**	55 (94.8) 3 (5.2)	**0.005**
*MALAT1* (rs3200401)	CC	175 (71.4)	82 (70.7)	0.823	19 (65.5)	0.773
	CT	61 (24.9)	31 (26.7)		9 (31)	
	TT	9 (3.7)	3 (2.6)		1 (3.4)	
	In total:	245 (100)	116 (100)		29 (100)	

Bold p-values indicate statistical significance after Bonferroni correction (p < 0.016).

Binary logistic regression analysis revealed that the G allele of *MALAT1* rs619586 increases the odds of developing PA with recurrence by 6.9-fold under the additive model (OR = 6.952, 95% CI: 1.475-32.775, p = 0.014) (**Table 11**).

**Table 11:** Binary logistic regression analysis of *MALAT1* (rs1194338, rs619586 and rs3200401) in the PA and control groups by PA recurrence.

*MALAT1* (1194338)				
PA without recurrence				
Model	Genotype/Allele	OR (95% CI)	*P*-value	AIC
Codominant	CA vs. CC AA vs. CC	0.808 (0.501-1.304) 0.509 (0.163-1.587)	0.383 0.244	455.338
Dominant	CA+AA vs. CC	0.763 (0.482-1.209)	0.249	453.979
Recessive	AA vs. CC+CA	0.548 (0.178-1.688)	0.294	454.107
Overdominant	CA vs. CC+AA	0.847 (0.528-1.360)	0.492	454.844
Additive	A	0.767 (0.522-1.129)	0.179	453.466
PA with recurrence				
Codominant	CA vs. CC AA vs. CC	1.365 (0.610-3.052) 1.289 (0.269-6.184)	0.449 0.751	188.482
Dominant	CA+AA vs. CC	1.353 (0.626-2.928)	0.442	186.487
Recessive	AA vs. CC+CA	1.136 (0.246-5.237)	0.870	187.049
Overdominant	CA vs. CC+AA	1.329 (0.606-2.911)	0.478	186.578
Additive	A	1.235 (0.675-2.261)	0.494	186.618
*MALAT1* (rs619586)				
PA without recurrence				
Model	Genotype/Allele	OR (95% CI)	*P*-value	AIC
Codominant	AG vs. AA GG vs. AA	2.764 (0.728-10.493) -	0.135 -	450.533
Dominant	AG+GG vs. AA	3.869 (1.110-13.493)	0.034	450.588
Recessive	GG vs. AA+AG	–	–	450.756
Overdominant	AG vs. AA+GG	2.714 (0.715-10.301)	0.142	453.174
Additive	G	3.620 (1.173-11.177)	0.025	449.315
PA with recurrence				
Codominant	AG vs. AA GG vs. AA	6.952 (1.475-32.775) -	0.014	184.058
Dominant	AG+GG vs. AA	6.952 (1.475-32.775)	0.014	182.058
Recessive	GG vs. AA+AG	–	–	182.058
Overdominant	AG vs. AA+GG	6.952 (1.475-32.775)	0.014	182.058
Additive	G	6.952 (1.475-32.775)	**0.014**	**182.058**
*MALAT1*(rs3200401)				
PA without recurrence				
Model	Genotype/Allele	OR (95% CI)	*P*-value	AIC
Codominant	CT vs. CC TT vs. CC	1.085 (0.654-1.798) 0.711 (0.188-2.697)	0.753 0.616	456.919
Dominant	CT+TT vs. CC	1.037 (0.637-1.686)	0.885	455.300
Recessive	TT vs. CC+CT	0.696 (0.185-2.621)	0.592	455.018
Overdominant	CT vs. CC+TT	1.100 (0.665-1.819)	0.710	455.183
Additive	T	0.988 (0.652-1.496)	0.954	455.317
PA with recurrence				
Codominant	CT vs. CC TT vs. CC	1.359 (0.584-3.163) 1.023 (0.123-8.522)	0.477 0.983	188.580
Dominant	CT+TT vs. CC	1.316 (0.583-2.971)	0.509	186.648
Recessive	TT vs. CC+CT	0.937 (0.114-7.669)	0.951	187.071
Overdominant	CT vs. CC+TT	1.357 (0.587-3.139)	0.475	186.580
Additive	T	1.202 (0.612-2.360)	0.594	186.799

OR, odds ratio; CI, confidence interval; AIC, Akaike information criteria; p-value, significance level (statistically significant when p < 0.016).

Bold p-values indicate statistical significance after Bonferroni correction (p < 0.016). Bold AIC values indicate the best-fitting model (AIC).

### 3.5 Ki-67 labeling index

76 PA tissue samples were analyzed. The Ki-67 Labeling Index (LI) was evaluated in 43 females (56.6%) and 33 males (43.4%). The results revealed no statistically significant differences in the Ki-67 LI between females and males (p = 0.079).

Immunohistochemistry for Ki-67 revealed an LI < 1% in 23.7% of patients with PA, a Ki-67 LI 1% in 7.9%, and a Ki-67 LI > 1% in 68.4% of patients. Further analyses revealed no statistical significance concerning tumor size (p=0.199), invasiveness (p=0.160), activity (p=0.207), or recurrence (p=0.853) (**Supplementary Table S5**). The analysis of the Ki-67 LI with the indicated genetic variations (*MALAT1* rs1194338, rs619586, and rs3200401) also revealed no statistically significant results (**SupplementaryTable S6**).

### 3.6 p53 analysis in PA tissues

62 PA tissue samples were analyzed for p53. The p53 was evaluated in 32 women (51.6%) and 30 men (48.4%). The results revealed no statistically significant differences in the p53 H-score between women and men (p = 0.916). Immunohistochemistry for p53 revealed that macroadenomas had statistically significantly higher p53 H-score compared to the microadenomas (median (IQR): 27.34 (32.16) vs. 16 (18.33), p = 0.047). Further analyses revealed no statistical significance regarding the PA invasiveness, activity, or recurrence (**Table 12**).

**Table 12:** Associations of clinical features of PA with p53 H-score.

PA subgroups	p53 H score median (IQR)	*P*-value*
Micro PA	16 (18.33)	**0.047**
Macro PA	27.34 (32.16)	
Non-invasive PA	22.66 (18.42)	0.490
Invasive PA	26.67 (31.72)	
Non-active PA	18.34 (23.65)	0.272
Active PA	27.99 (32.33)	
PA without recurrence	26.67 (32.58)	0.167
PA with recurrence	15.01 (17.41)	

*Mann-Whitney U test was used; PA – pituitary adenoma.

Bold p-value indicates statistical significance (p < 0.05).

**Figure 1:**
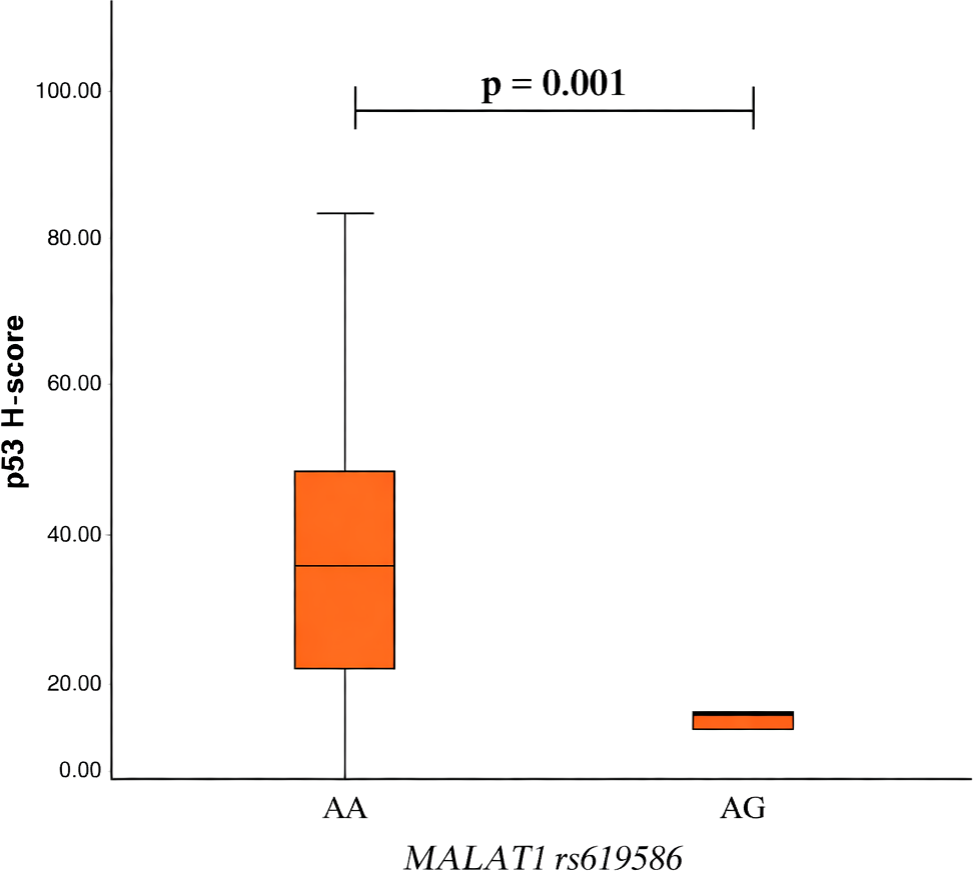
*MALAT1* rs619586 genotype associations with p53 H-score.

To assess the association of the *MALAT1* rs1194338, rs619586 and rs3200401 variants with p53, the p53 H-score was calculated in different genotype groups. We found no statistical significant results comparing *MALAT1* rs1194338 and rs3200401 genotypes with p53 H-score (**Supplementary Figure S1** and **Supplementary Figure S2**). Moreover, PA patients who had the *MALAT1* rs619586 AA genotype had statistically significantly higher p53 H-score than patients with AG genotype (median (IQR): 26.33 (28.91) vs. 9.67 (5.84), p = 0.001, respectively) (Mann-Whitney U test was used) (**Figure 1**).

### 3.7 Correlation between Ki-67 and p53.

**Figure 2:**
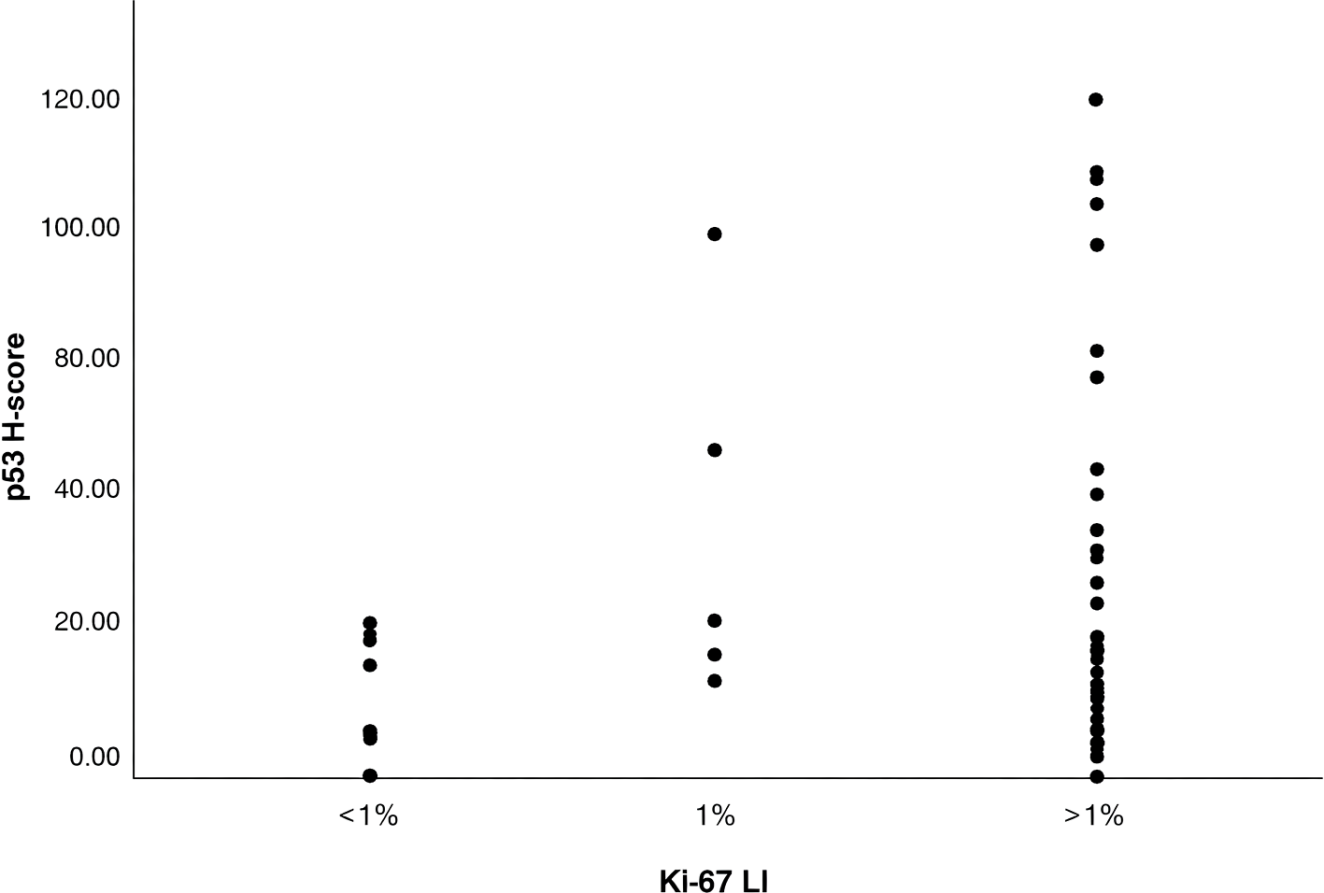
Correlation between Ki-67 LI and p53 H-score.

A nonparametric Spearman’s rank-order correlation was performed to assess the relationship between Ki-67 LI and p53 H-score. The results demonstrated a moderate, statistically significant positive correlation between Ki-67 LI and p53 expression (Spearman’s ρ=0.268, p=0.035, n=62). The 95% CI for the correlation coefficient ranged from 0.012 to 0.491. These findings suggest that increased proliferative activity, as measured by Ki-67, is associated with higher p53 expression in the studied samples. The scatter plot categorizes Ki-67 LI into <1%, 1%, and >1%. It shows that higher Ki-67 levels are generally associated with higher p53 H-scores, particularly in the >1% group, where more variability and elevated H-scores are evident (**Figure 2**).

## 4 Discussion

This study aimed to investigate the involvement of lncRNA *MALAT1* gene variants in the pathology of pituitary adenoma and their potential clinical relevance.

Long non-coding RNAs (lncRNAs) have emerged as important regulators of gene expression, chromatin structure, and cellular homeostasis. In pituitary adenomas, lncRNAs modulate fundamental biological processes such as cell proliferation, apoptosis, differentiation and tumour progression, thereby influencing tumour phenotype and aggressiveness (26, 27).

Also known as nuclear enrichment autosomal transcript 2 (*NEAT2*), *MALAT1* was initially identified through subtractive hybridization as one of the transcripts most significantly overexpressed in metastatic non-small cell lung cancer tissues (26). Since then, *MALAT1* has become one of the most extensively studied lncRNAs, with accumulating evidence suggesting its multifaceted roles in pituitary adenoma biology (28). The available literature supports the concept that *MALAT1* may contribute to tumorogenesis and progression in pituitary adenomas through mechanisms similar to those described in other cancers, such as promoting proliferation, angiogenesis, apoptosis, and epithelial-mesenchymal transition (EMT) (26, 29, 30).

In recent years, studies investigating the role of lncRNA *MALAT1* in the pathogenesis of pituitary adenoma development have shown inconsistent results. Li and colleagues evaluated *MALAT1* alongside *MEG3* and *HOTAIR* expression in non-functioning pituitary adenoma (NFPAs) and reported no significant difference in *MALAT1* expression between tumor and normal pituitary tissue, nor did they correlate with clinicopathological parameters (31). It seems that MALAT1 may exert an anti-cancer effect in NFPAs (32). Lu et al. examined growth-hormone-secreting pituitary adenomas (GHPA) and reported that high *H19* expression was associated with tumour invasion, whereas *MALAT1* expression did not differ significantly between invasive and non-invasive GHPA (33). Recent work by Ghafouri-Fard et al. analysed lncRNAs in pituitary adenoma tissues and observed a strong positive correlation between PVT1 and *MALAT1*, suggesting that interactions between lncRNAs may contribute to tumour pathogenesis and highlight the complex regulatory networks in which *MALAT1* participates (34).

While several *MALAT1* SNVs, including rs619586, have been investigated in lung, colorectal, hepatocellular and other cancers, the evidence that these variants influence risk or progression, often through changes in *MALAT1* expression and downstream oncogenic pathways, is inconsistent (35). To date, there is currently no established evidence of direct or consistent association between *MALAT1* SNVs and neuroendocrine tumors, particularly PAs, susceptibility or behavior (29, 36).

Our study is the first to demonstrate that the *MALAT1* rs6198586 *G* allele is significantly more frequent in pituitary adenoma patients compared to controls and is associated with tumour aggressiveness, including invasiveness and recurrence. These results contrast with most other reports in other diseases, where the rs619586 allele tends to be protective. It is important to note that rs619586 is located within a non-coding region of the *MALAT1* gene, and direct functional evidence demonstrating its impact on *MALAT1* expression, splicing, or RNA-binding properties in pituitary cells is currently lacking (28).

Although functional studies in other tumor types have linked rs619586 to altered *MALAT1* expression and tumor-related phenotypes, these effects appear to be tissue-specific (18, 21). Therefore, the present findings should be interpreted as evidence of genetic association rather than direct mechanistic causality. Functional studies in pituitary-derived models will be required to elucidate the molecular mechanisms underlying the observed associations.

For example, a meta-analysis of nine case-control studies involving several cancer types found that the rs619586 G allele was associated with reduced overall cancer risk in Asians (odds ratio ≈ 0.87 for the G vs. A allele) (37), and a case-control study of papillary thyroid cancer (PTC) demonstrated that the rs619586 G allele was a protective factor (OR ≈ 0.76) while decreasing.

*MALAT1* expression, suppressed cell proliferation and increased apoptosis (18). Similarly, in studies of cerebral tumors, for example meningioma, the rs619586 A>G variant lowers *MALAT1* expression and reduces tumor invasiveness (19). In contrast, in oral squamous cell carcinoma (OSCC), the rs619586 AG/GG genotype was associated with higher tumour stage and larger tumours, especially in patients with a betel-nut chewing habit (21). Together, these findings illustrate the tissue-specific nature of rs619586, it is protective or neutral in most cancers, yet linked to aggressiveness in OSCC and, as our data show, in pituitary adenoma.

The current literature suggests that rs3200401 and rs1194338 have cancer type-specific effects, in some cases, and in others are associated with aggressive disease. In advanced lung adenocarcinoma patients, the rs3200401 T allele was associated with better survival (38). Qu Y et al. reported that the T allele increases the risk of esophageal squamous cell carcinoma (39). A Taiwanese prostate cancer cohort showed that male patients with at least one rs1194338 A allele had more than a threefold increased risk of lymph-node metastasis (40). In our study, we did not observe any significant associations between these *MALAT1* variants and pituitary adenoma characteristics.

A high Ki-67 LI is generally interpreted as evidence of rapid proliferation and potential invasiveness into surrounding structures (41). Several Ki-67 cut-off values have been proposed to stratify aggressiveness in pituitary adenomas, ranging from 1,5% to 4% (42). Some studies have linked higher Ki−67 indices to invasiveness or recurrence, whereas others have not.

A large retrospective analysis of pituitary adenomas found no significant differences in Ki-67 LI with respect to sex, tumor type, diameter, or invasiveness, although the same study reported an association between higher Ki-67 LI and recurrence (43). In a review involving 28 studies on Ki- 67, 18 studies reported high Ki-67 expression in recurrent adenomas, while the other 10 studies showed no correlation (44). A subsequent meta−analysis concluded that a Ki−67 ≥ 3% warrants closer postoperative surveillance, as these tumors were more likely to recur; however, Ki-67 is not an independent predictor of tumor recurrence. Moreover, factors such as tumor subtype, extent of surgical resection, cavernous sinus invasion and hormonal activity often exert a stronger influence on recurrence than Ki−67 alone (45).

Our findings that Ki-67 LI did not correlate with tumor size, invasiveness, hormonal activity or recurrence align with those studies showing limited prognostic utility of Ki-67. Also there were no significant results between Ki-67 Li and *MALAT1* genetic variations.

Normal pituitary tissue expresses little p53, and most pituitary adenomas show only minimal or focal p53 staining. Earlier studies have suggested that tumors with high p53 expression exhibit more frequent tumor progression and cavernous sinus invasion, but they did not assess recurrence (46, 47). Oliveira et al. subsequently reported no correlation between p53 expression and PA recurrence. In their study of 148 pituitary adenomas, only 1.3% were p53-positive, indicating that p53 is insufficient as a routine marker of recurrence (48). Our study demonstrated that macroadenomas had significantly higher p53 H-scores than microadenomas, however, p53 expression was not associated with invasiveness, hormonal activity or recurrence. Also, we found that patients carrying the *MALAT1* rs619586 AA genotype had higher p53 H-scores than those with AG genotype (median 26.33 vs. 9.67, p = 0,001). Moreover, p53-positive adenomas generally display a higher Ki-67 compared with p53-negative tumors, which is consistent with previous observations (47). Although p53 H-scores were higher in tumors from patients with the rs619586 AA genotype, this does not contradict the association of the rs619586 G allele with more aggressive clinical features. In pituitary adenomas, increased p53 immunoreactivity often reflects accumulation of dysfunctional or stabilized p53 protein and does not consistently correlate with invasion, recurrence, or proliferative activity (47). Therefore, p53 expression should be interpreted as a context-dependent cellular response rather than a direct indicator of tumor aggressiveness (46, 49). The discordant directions observed between p53 expression and clinical aggressiveness further support the notion that rs619586-associated tumor behavior is unlikely to be mediated directly through p53 signaling.

Despite the valuable insights gained through this exploratory study, limitations undoubtedly exist. Future studies could evaluate these variants in larger PA cohorts, in diverse ethnic groups and functional assays to determine whether they influence tumour susceptibility or behaviour, and functional assays should clarify how these variants modulate *MALAT1* expression or its interactions with downstream targets.

Although rs619586 was consistently associated with PA risk and several aggressive clinical features in the present cohort, independent replication in an external population has not yet been performed. Therefore, rs619586 should be regarded as a potential or candidate genetic marker rather than a validated biomarker. Replication in independent, geographically distinct cohorts will be required to confirm these findings and to determine their generalizability.

## 5 Limitations

Although age and sex were matched between cases and controls, multivariable logistic regression models incorporating clinical factors such as hormone excess, medication use, and treatment history were not applied in the present analysis. This study was designed primarily to evaluate genetic associations, and many clinical variables are intrinsically related to tumor phenotype and post-diagnostic management rather than baseline genetic risk. In addition, the low frequency of certain variants limited the feasibility of stable multivariable modeling.

Given the low number of rs619586 G-allele carriers, odds ratio estimates derived from standard logistic regression, particularly in subgroup analyses, may be affected by sparse data bias and should therefore be interpreted with caution. Future studies in larger cohorts will be required to obtain more stable effect estimates and to delineate the independent and combined effects of genetic and clinical factors on pituitary adenoma risk and behavior.

In addition, no statistically significant associations were observed for p53 H-score or Ki-67 labeling index in several analyses. This may be attributable to the heterogeneous and context-dependent expression of these markers in PAs, as well as limited statistical power after subgroup stratification. Moreover, p53 and Ki-67 reflect downstream cellular responses and proliferative activity, which may not directly mirror underlying genetic susceptibility. Larger studies integrating comprehensive clinicopathological data will be required to better define their relationship with *MALAT1* genetic variants.

Another limitation of this study is the lack of ancestry informative markers, which precludes formal testing for subtle population stratification. However, the relatively homogeneous ethnic background of the cohort and Hardy–Weinberg equilibrium in the control group mitigate, though do not eliminate, this concern.

The relatively small recurrence subgroup and the inability to fully distinguish true biological recurrence from regrowth of residual tumor tissue represent additional limitations of the recurrence-related analyses.

Finally, tumor invasiveness was assessed using radiological Knosp grading, which, although clinically standard, has limited sensitivity for detecting microscopic invasion. Integration of surgical and histopathological validation in future studies may therefore improve predictive accuracy.

## 6 Conclusions

This study highlights the potential role of *MALAT1* genetic variants, particularly rs619586, in the susceptibility and clinical behavior of PAs. The rs619586 G allele was found to be significantly more frequent among PA patients compared to controls and remained associated with several aggressive tumor features, including micro- and macroadenoma formation, invasiveness, and recurrence, even after Bonferroni correction. These findings suggest that rs619586 may serve as a molecular biomarker linked to PA development and progression. In addition, a significant association between *MALAT1* rs619586 genotypes and p53 expression, along with a positive correlation between p53 and Ki-67, further supports the interplay between genetic and proliferative.

factors in PA pathophysiology. Although other investigated variants (rs1194338 and rs3200401) did not show significant associations, the overall results underscore the importance of lncRNA *MALAT1* in pituitary tumorigenesis. Future studies involving larger and more diverse cohorts, as well as functional analyses, are warranted to validate these associations and elucidate the molecular mechanisms linking *MALAT1* dysregulation to PA aggressiveness and recurrence.

## Data Availability

The original contributions presented in the study are included in the article/**Supplementary Material**. Further inquiries can be directed to the corresponding author/s.
