# Supplementary material for: MALAT1 rs619586 as a potential genetic marker of pituitary adenoma susceptibility and aggressiveness
Source: Front Endocrinol (Lausanne). 2026 Feb 12;17:1748441. doi: 10.3389/fendo.2026.1748441 (PMC12935662; doi:10.3389/fendo.2026.1748441)
Supplement: Supplementary file 1 [file DataSheet1.docx]

**Supplementary Materials**

**Table S1. Distributions of *MALAT1* rs1194338, rs619586, and rs3200401 genotypes and alleles in female patients with PA and the control group**

| **Gene** | **Genotype/Allele** | **PA group females (n=80)**  **n (%)** | **Control group**  **females (n=135)**  **n (%)** | ***p*-Value** |
| --- | --- | --- | --- | --- |
| *MALAT1*  (rs1194338) | CC | 46 (57.5) | 79 (58.5) | 0.732 |
|  | CA | 30 (37.5) | 46 (34.1) |  |
|  | AA | 4 (5) | 10 (7.4) |  |
|  | In total: | 80 (100) | 135 (100) |  |
|  | Allele:  C  A | 122 (76.3)  38 (23.7) | 204 (75.6)  66 (24.4) | 0.870 |
| *MALAT1*  (rs619586) | AA | 75 (93.8) | 133 (98.5) | 0.057 |
|  | AG | 5 (6.3) | 2 (1.5) |  |
|  | GG | 0 (0) | 0 (0) |  |
|  | In total: | 80 (100) | 135 (100) |  |
|  | Allele:  A  G | 155 (96.9)  5 (3.1) | 268 (99.3)  2 (0.7) | 0.058 |
|  | CC | 52 (65) | 93 (68.9) | 0.841 |
|  | CT | 24 (30) | 36 (26.7) |  |
| *MALAT1* | TT | 4 (5) | 6 (4.4) |  |
| (rs3200401) | In total: | 80 (100) | 135 (100) |  |
|  | Allele:  C  T | 128 (80)  32 (20) | 222 (82.2)  48 (17.8) | 0.567 |
|  |  |  |  |  |
|  |  |  |  |  |

**Table S2. Binary logistic regression analysis of *MALAT1* rs1194338, rs619586, and rs3200401 in female patients with PA and the control group**

| ***MALAT1* (**rs1194338**)** | | | | |
| --- | --- | --- | --- | --- |
| **Model** | **Genotype/Allele** | **OR (95% CI)** | ***p*-Value** | **AIC** |
| Codominant | CA vs. CC  AA vs. CC | 1.120 (0.623-2.013)  0.687 (0.204-2.316) | 0.705  0.545 | 287.187 |
| Dominant | CA+AA vs. CC | 1.043 (0.596-1.826) | 0.884 | 285.805 |
| Recessive | AA vs. CC+CA | 0.658 (0.199-2.171) | 0.492 | 285.330 |
| Overdominant | CA vs. CC+AA | 1.161 (0.653-2.065) | 0.612 | 285.569 |
| Additive | A | 0.964 (0.615-1.512) | 0.873 | 285.800 |
| ***MALAT1* (**rs619586**)** | | | | |
| **Model** | **Genotype/Allele** | **OR (95% CI)** | ***p*-Value** | **AIC** |
| Codominant | AG vs. AA  GG vs. AA | 4.433 (0.840-23.411)  - | 0.079  - | 284.336 |
| Dominant | AG+GG vs. AA | 4.433 (0.840-23.411) | 0.079 | 282.336 |
| Recessive | GG vs. AA+AG | - | - | 285.826 |
| Overdominant | AG vs. AA+GG | 4.433 (0.840-23.411) | 0.079 | 282.336 |
| Additive | G | 4.433 (0.840-23.411) | 0.079 | 282.336 |
|  | ***MALAT*** | ***1*** (rs3200401) |  |  |
| **Model** | **Genotype/Allele** | **OR (95% CI)** | ***p*-Value** | **AIC** |
| Condominant | CT vs. CC  TT vs. CC | 1.192 (0.643-2.212)  1.192 (0.322-4.418) | 0.577  0.792 | 287.482 |
| Dominant | CT+TT vs. CC | 1.192 (0.663-2.143) | 0.557 | 285.482 |
| Recessive | TT vs. CC+CT | 1.132 (0.309-4.138) | 0.852 | 285.791 |
| Overdominant | CT vs. CC+TT | 1.179 (0.639-2.173) | 0.599 | 285.550 |
| Additive | T | 1.144 (0.709-1.847) | 0.582 | 285.524 |

OR: odds ratio; CI: confidence interval; AIC: Akaike information criteria; p-value: significance level (statistically significant when p < 0.05).

**Table S3. Binary logistic regression analysis of *MALAT1* rs1194338, rs619586, and rs3200401 in male patients with PA and control groups**

| ***MALAT1* (**rs1194338**)** | | | | |
| --- | --- | --- | --- | --- |
| **Model** | **Genotype/Allele** | **OR (95% CI)** | ***p*-Value** | **AIC** |
| Codominant | CA vs. CC  AA vs. CC | 0.677 (0.345-1.329)  0.587 (0.109-3.157) | 0.257  0.535 | 233.354 |
| Dominant | CA+AA vs. CC | 0.667 (0.348-1.277) | 0.222 | 231.381 |
| Recessive | AA vs. CC+CA | 0.667 (0.126-3.539) | 0.634 | 232.661 |
| Overdominant | CA vs. CC+AA | 0.697 (0.357-1.361) | 0.291 | 231.764 |
| Additive | A | 0.708 (0.403-1.243) | 0.230 | 231.411 |
| ***MALAT1* (**rs619586**)** | | | | |
| **Model** | **Genotype/Allele** | **OR (95% CI)** | ***p*-Value** | **AIC** |
| Codominant | AG vs. AA  GG vs. AA | 2.700 (0.439-16.611)  - | 0.284  - | 229.720 |
| Dominant | AG+GG vs. AA | 4.500 (0.847-23.903) | 0.078 | 229.366 |
| Recessive | GG vs. AA+AG | - | - | 229.898 |
| Overdominant | AG vs. AA+GG | 2.613 (0.425-16.066) | 0.300 | 231.797 |
| Additive | G | 3.922 (0.903-17.037) | 0.068 | 228.324 |
|  | ***MALAT*** | ***1*** (rs3200401) |  |  |
| **Model** | **Genotype/Allele** | **OR (95% CI)** | ***p*-Value** | **AIC** |
| Condominant | CT vs. CC  TT vs. CC | 1.071 (0.521-2.201)  - | 0.852  - | 232.048 |
| Dominant | CT+TT vs. CC | 0.956 (0.471-1.943) | 0.902 | 232.884 |
| Recessive | TT vs. CC+CT | - | - | 230.082 |
| Overdominant | CT vs. CC+TT | 1.110 (0.541-2.279) | 0.776 | 232.818 |
| Additive | T | 0.855 (0.446-1.636) | 0.635 | 232.671 |

OR: odds ratio; CI: confidence interval; AIC: Akaike information criteria; p-value: significance level (statistically significant when p < 0.05).

**Table S4. Binary logistic regression analysis of *MALAT1* rs1194338, rs619586, and rs3200401 in the PA and control groups by PA activity**

| ***MALAT1* (**1194338**)** | | | | |
| --- | --- | --- | --- | --- |
| **Non-active PA** | | | | |
| **Model** | **Genotype/Allele** | **OR (95% CI)** | ***p*-Value** | **AIC** |
| Codominant | CA vs. CC  AA vs. CC | 0.775 (0.429-1.402)  0.439 (0.097-1.996) | 0.400  0.287 | 323.686 |
| Dominant | CA+AA vs. CC | 0.725 (0.409-1.284) | 0.270 | 322.260 |
| Recessive | AA vs. CC+CA | 0.479 (0.107-2.150) | 0.337 | 322.407 |
| Overdominant | CA vs. CC+AA | 0.818 (0.455-1.472) | 0.504 | 323.046 |
| Additive | A | 0.730 (0.449-1.186) | 0.203 | 321.806 |
| **Active PA** | | | | |
| Codominant | CA vs. CC  AA vs. CC | 1.016 (0.593-1.742)  0.823 (0.260-2.601) | 0.953  0.740 | 363.804 |
| Dominant | CA+AA vs. CC | 0.987 (0.589-1.655) | 0.961 | 361.930 |
| Recessive | AA vs. CC+CA | 0.818 (0.263-2.540) | 0.728 | 361.807 |
| Overdominant | CA vs. CC+AA | 1.033 (0.608-1.757) | 0.903 | 361.918 |
| Additive | A | 0.963 (0.632-1.468) | 0.861 | 361.902 |
| ***MALAT1* (**rs619586**)** | | | | |
| **Non-active PA** | | | | |
| **Model** | **Genotype/Allele** | **OR (95% CI)** | ***p*-Value** | **AIC** |
| Codominant | AG vs. AA  GG vs. AA | 3.951 (0.961-16.249)  - | 0.057  - | 318.990 |
| Dominant | AG+GG vs. AA | 4.939 (1.287-18.945) | 0.020 | 318.264 |
| Recessive | GG vs. AA+AG | - | - | 320.388 |
| Overdominant | AG vs. AA+GG | 3.887 (0.945-15.981) | 0.060 | 320.176 |
| Additive | G | 4.633 (1.312-16.358) | 0.017 | 317.378 |
| **Active PA** | | | | |
| Codominant | AG vs. AA  GG vs. AA | 3.257 (0.795-13.342)  - | 0.101  - | 359.537 |
| Dominant | AG+GG vs. AA | 4.071 (1.066-15.552) | 0.040 | 357.812 |
| Recessive | GG vs. AA+AG | - | - | 359.100 |
| Overdominant | AG vs. AA+GG | 3.213 (0.785-13.161) | 0.105 | 359.424 |
| Additive | G | 3.900 (1.123-13.546) | 0.032 | 356.990 |
|  | ***MALAT1*** | (rs3200401) |  |  |
| **Non-active PA** | | | | |
| **Model** | **Genotype/Allele** | **OR (95% CI)** | ***p*-Value** | **AIC** |
| Codominant | CT vs. CC  TT vs. CC | 0.717 (0.366-1.407)  0.374 (0.046-3.020) | 0.334  0.356 | 323.611 |
| Dominant | CT+TT vs. CC | 0.673 (0.351-1.292) | 0.234 | 322.017 |
| Recessive | TT vs. CC+CT | 0.403 (0.050-3.243) | 0.393 | 322.583 |
| Overdominant | CT vs. CC+TT | 0.740 (0.378-1.449) | 0.380 | 322.700 |
| Additive | T | 0.683 (0.385-1.213) | 0.194 | 321.681 |
| **Active PA** | | | | |
| Codominant | CT vs. CC  TT vs. CC | 1.581 (0.909-2.748)  1.190 (0.310-4.567) | 0.104  0.799 | 361.349 |
| Dominant | CT+TT vs. CC | 1.531 (0.899-2.606) | 0.117 | 359.517 |
| Recessive | TT vs. CC+CT | 1.035 (0.273-3.921) | 0.960 | 361.930 |
| Overdominant | CT vs. CC+TT | 1.566 (0.906-2.708) | 0.108 | 359.412 |
| Additive | T | 1.353 (0.869-2.108) | 0.181 | 360.186 |

OR: odds ratio; CI: confidence interval; AIC: Akaike information criteria; p-value: significance level (statistically significant when p < 0.05).

**Table S5. Ki-67 LI, considering the characteristics of PA**

| **Characteristics** | | **Ki-67 LI** | | | | ***p*-Value** |
| --- | --- | --- | --- | --- | --- | --- |
|  |  | **<1%** | **1%** | | **>1%** |  |
| **Tumour size** | Micro PA (n=25) (%) | 9 (36) | | 2 (8) | 14 (56) | 0.199 |
|  | Macro PA (n=51) (%) | 9 (17.6) | | 4 (7.8) | 38 (74.5) |  |
| **Invasiveness** | Non-invasive PA (n=36) (%) | 11 (30.6) | | 1 (2.8) | 24 (66.7) | 0.160 |
|  | Invasive PA (n=40) (%) | 7 (17.5) | | 5 (12.5) | 28 (70) |  |
| **Activeness** | Non-active PA (n=37) (%) | 8 (21.6) | | 1 (2.7) | 28 (75.7) | 0.207 |
|  | Active PA (n=39) (%) | 10 (25.6) | | 5 (12.8) | 24 (61.5) |  |
| **Recurrence** | PA without recurrence (n=56) (%) | 13 (23.2) | | 5 (8.9) | 38 (67.9) | 0.853 |
|  | PA with recurrence (n=20) (%) | 5 (25) | | 1 (5) | 14 (70) |  |

**Table S6. Ki-67 labeling index associations with *MALAT1* rs1194338, rs619586 and rs3200401**

| **Gene, SNV** | **Genotype/Allele** | **Ki-67 LI** | | ***p*-Value** |
| --- | --- | --- | --- | --- |
|  |  | **≤1%** | **>1%** |  |
| *MALAT1* rs1194338 | CC | 13 (54.2) | 35 (67.3) | 0.375 |
|  | CA | 11 (45.8) | 16 (30.8) |  |
|  | AA | 0 (0) | 1 (1.9) |  |
|  | In total: | 24 (100) | 52 (100) |  |
|  | Allele:  C  A | 37 (77.1)  11 (22.9) | 86 (82.7)  18 (17.3) | 0.413 |
| *MALAT1* rs619586 | AA | 21 (87.5) | 48 (92.3) | 0.485 |
|  | AG | 3 (12.5) | 3 (5.8) |  |
|  | GG | 0 (0) | 1 (1.9) |  |
|  | In total: | 24 (100) | 52 (100) |  |
|  | Allele:  A  G | 45 (93.8)  3 (6.2) | 99 (95.2)  5 (4.8) | 0.711 |
| *MALAT1*  rs3200401 | CC | 18 (75) | 36 (69.2) | 0.243 |
|  | CT | 5 (20.8) | 16 (30.8) |  |
|  | TT | 1 (4.2) | 0 (0) |  |
|  | In total: | 24 (100) | 52 (100) |  |
|  | Allele:  C  T | 41 (85.4)  7 (14.6) | 88 (84.6)  16 (15.4) | 0.898 |


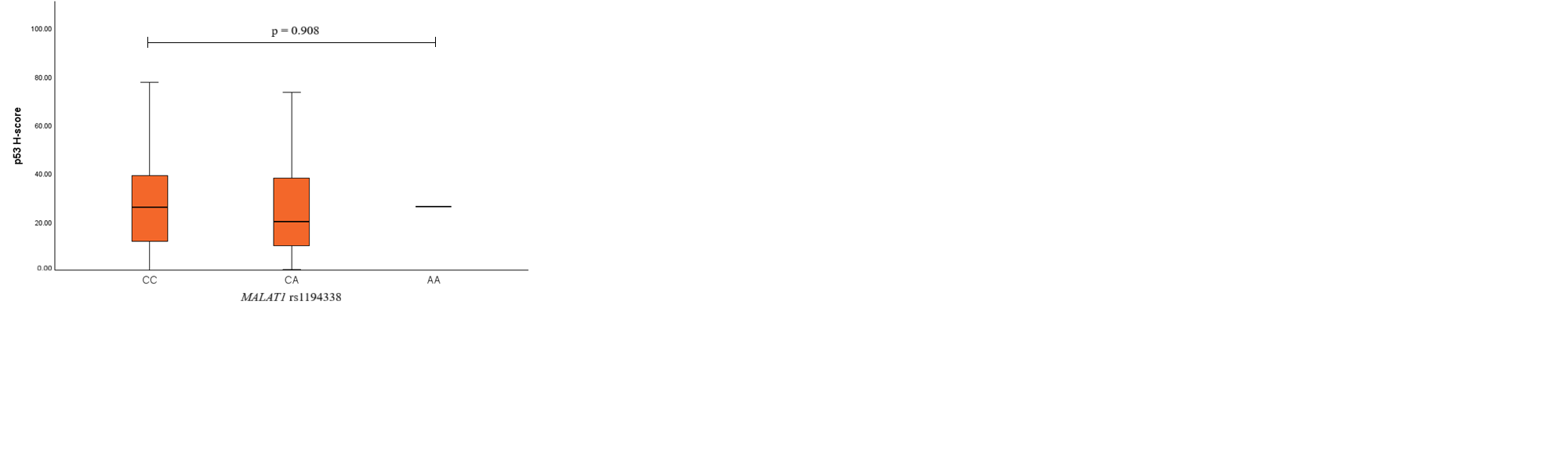


**Figure S1. *MALAT1* rs1194338 genotype associations with p53 H-score**


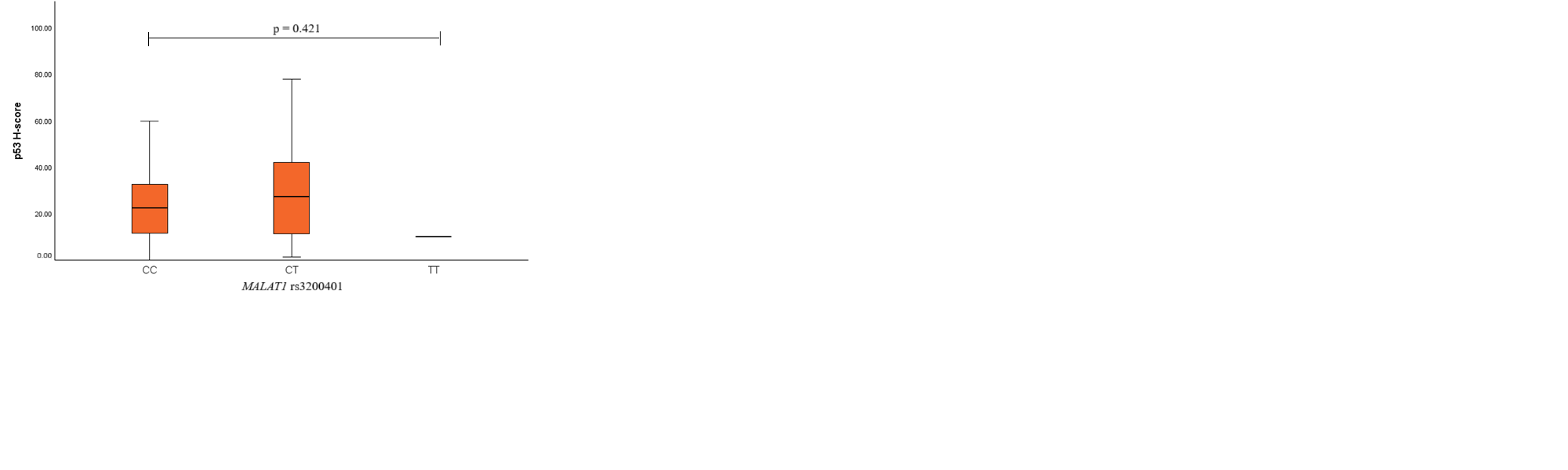


**Figure S2. *MALAT1* rs3200401 genotype associations with p53 H-score**
